# Exploring pain-motor dynamics: preliminary insights through exploration of descending inhibition and corticospinal excitability

**DOI:** 10.3389/fpain.2026.1772887

**Published:** 2026-04-15

**Authors:** Arnaud Duport, Hervé Devanne, Guillaume Léonard

**Affiliations:** 1 Research Centre on Aging, CIUSSS de l'Estrie-CHUS, Sherbrooke, QC, Canada; 2 Faculty of Medicine and Health Sciences, Université de Sherbrooke, Sherbrooke, QC, Canada; 3 Univ. Littoral Côte d'Opale, Univ. Artois, Univ. Lille, ULR 7369 - URePSSS - Unité de Recherche Pluridisciplinaire Sport Santé Société, Calais, France

**Keywords:** conditioned pain modulation, corticospinal excitability, experimental pain, input-output curves, kinesiophobia, pain catastrophizing, transcranial magnetic stimulation

## Abstract

**Introduction:**

Previous studies have linked endogenous pain modulation and corticospinal excitability (CSE), but methodological limitations and overlooking psychological factors may have constrained interpretations. This study aimed to evaluate the interaction between CSE excitability in different muscles and endogenous pain modulation, and to determine whether kinesiophobia and pain catastrophizing modulate this interaction.

**Methods:**

Twenty-one pain-free participants completed questionnaires on kinesiophobia and pain catastrophizing. Conditioned pain modulation (CPM) was used to assess endogenous pain modulation. Transcranial magnetic stimulation was used to assess the pain-induced modulation in CSE excitability, focusing on slope, S_50_ and the maximum response parameters of input-output curves (plateau) for the anterior deltoid (AD) and first dorsal interosseous (FDI) muscles, first in pain-free then in painful condition induced by the application of capsaicin cream to the shoulder.

**Results:**

A significant correlation was found between the plateau of the AD input-output curves measured at baseline (pain-free condition) and CPM responses (r_S_ = .56, *p* = 0.01), suggesting that higher maximal corticospinal output is associated with more effective endogenous pain modulation. Pain-induced changes in FDI slope and CPM responses were strongly correlated (r_S_ = -.75, *p* < 0.001), indicating that individuals with the most effective endogenous pain inhibition mechanisms were those with the greatest increase in CSE. Finally, kinesiophobia was found to alter the association between pain-induced changes in CSE in AD (S_50_ shift) and CPM response, shedding new light on the influence of psychological factors on pain-induced CSE alterations and their link with descending pain inhibition.

**Discussion:**

These findings underscore the complex interplay between corticospinal projections, pain modulation, and psychological factors, reinforcing the need for further investigation.

## 1 Introduction

The investigation of the interplay between pain and the motor system has gained considerable interest in the past few years (1–3). Transcranial magnetic stimulation (TMS) is a valuable tool for studying this interaction, having been utilized extensively to study corticospinal pathways (4). For pain researchers, this non-invasive approach can be used to stimulate the human cortex and assess the functional integrity of corticospinal and corticobulbar motor pathways under painful and non-painful conditions, leading to a better understanding of the effects of pain on the motor system (5–7).

Marked progress has also been made in recent years in our understanding of endogenous pain control mechanisms (8, 9). These pain modulating systems can be assessed using conditioned pain modulation paradigms (CPM), reflecting the ability of the central nervous system to inhibit pain signals through intricate neurophysiological mechanisms (10). CPM is believed to engage descending pain inhibitory pathways and the release of various molecules (including endogenous opioids), which dampen the transmission of pain signals in the central nervous system, in response to a noxious conditioning stimulation (9, 11, 12).

Two recent studies by Granovsky et al. and Martel et al. have explored the connection between corticospinal and pain modulation systems (1, 2). Their results suggest possible associations between CPM responses and corticospinal excitability, with individuals with higher corticospinal excitability showing greater CPM responses (1, 2). These observations open new perspectives in our understanding of the interaction between pain and the motor system and suggest that descending corticospinal and descending pain modulating circuits could be intertwined. However, these two studies present a number of limitations, such as the use of TMS input-output (IO) curves constructed with a restricted range of intensities and the oversight in considering psychological factors.

One of these important psychological factors, kinesiophobia, is characterized by a fear of movement or physical activity based on the belief that it may cause pain or injury (13, 14). Kinesiophobia has been shown to influence the effect of pain on corticospinal excitability (15–17), and plays a crucial role in the chronification of pain (14).

It is also important to point out that the studies of Granovsky et al. and Martel et al. concentrated solely on TMS measurements in a single condition, neglecting to evaluate fluctuations in these measurements in the absence and presence of pain (1, 2). Furthermore, given that changes in corticospinal excitability may be different for muscles far from the painful site than for muscles in the vicinity of the pain location (18), correlations between CPM and these changes in excitability could also differ. Indeed, a number of studies have shown that experimental pain typically decreases corticospinal excitability near the painful site (19–21), while effect on muscles distant from the induction site is more variable (20, 22), underscoring the need to investigate the relationship between CPM and corticospinal excitability changes at both locations.

A better understanding of how corticospinal excitability interacts with CPM and psychological factors such as kinesiophobia may help refine individual pain profiles and improve predictions of chronic pain vulnerability and rehabilitation outcomes. Understanding these multidimensional interactions could guide the development of personalized interventions aimed at restoring the balance between motor and pain inhibitory systems, for example by using neuromodulatory techniques (e.g., repetitive transcranial magnetic stimulation, transcranial direct current stimulation) or motor retraining to improve pain management and functional recovery. Ultimately, identifying neurophysiological and psychological markers associated with impaired descending inhibition and motor control could improve early detection of patients at risk for pain chronification, and inform mechanism-based therapeutic approaches.

This prospective within-subject experimental laboratory study in healthy volunteers aimed to replicate and extend the findings of Granovsky et al. and Martel et al. by investigating the association between descending motor pathway excitability (assessed using TMS IO curves in pain-free and pain conditions, both proximal and distal to the painful site) and descending pain inhibition. A further objective was to determine whether psychological factors such as kinesiophobia and pain catastrophizing can modulate these relationships.

## 2. Materials and methods

### 2.1 Participants and course of the study

**Figure 1:**
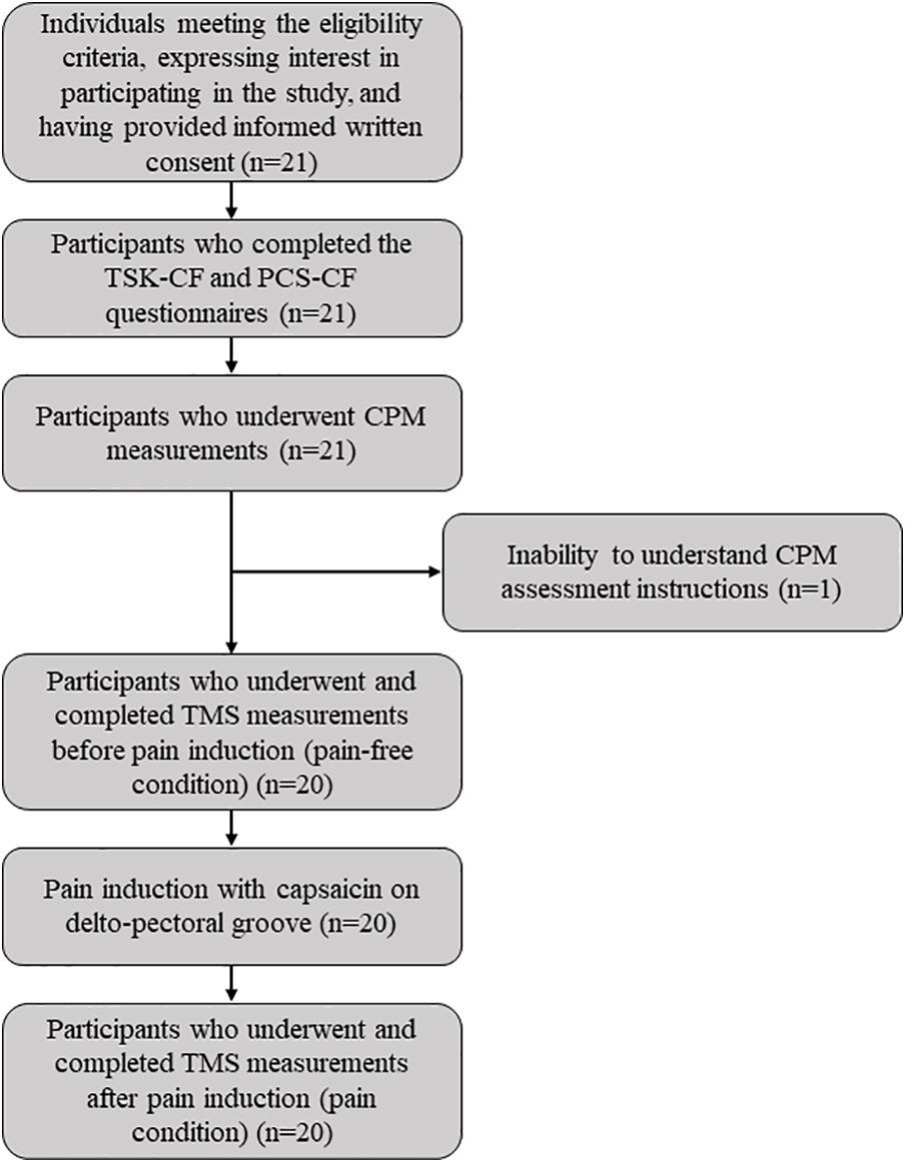
Participant flowchart.

In line with previous studies employing similar assessments and comparable experimental designs (3, 23), twenty-one pain-free individuals (10 females, 11 males) were recruited to take part in the experiment (Figure 1). To take part in the study, participants had to meet the following inclusion criteria: to be able to understand instructions, to abstain from tobacco and caffeine at least 2 h before data collection (24), and from short-acting analgesics (e.g., acetaminophen) at least 6 h before data collection. People meeting the following criteria were excluded: person living with a painful condition or suffering from chronic pain, presence of neurological disorders (e.g., stroke, epilepsy), contraindication to cold pressor test (e.g., Raynaud's syndrome), history of shoulder disorder, and contraindication to TMS (e.g., intracranial metal foreign bodies, hearing aids, or cochlear implants) (25).

Testing took place from February 5, 2022, to December 1, 2023 in the Research Centre on Aging (Sherbrooke, Canada). The experiment consisted of 2 sessions separated by 1 to 5 days to avoid CPM carryover effects (26). During the first session, participants were asked to complete kinesiophobia and pain catastrophizing questionnaires and then underwent an assessment of pain inhibitory mechanisms through the CPM paradigm. During the second session, the TMS measurements were performed to assess CSE using IO curves, initially under pain-free condition, and then after experimental pain induction with capsaicin on the delto-pectoral groove (painful condition). The two CSE assessments were separated by approximately 10 min. TMS measurements were collected both near the painful site (anterior deltoid) and far from the painful site (first dorsal interosseous). The study did not involve randomization, blinding, or control group.

The research protocol was approved by the ethics committee of the *CIUSSS de l'Estrie–CHUS* (Sherbrooke, QC, Canada; approval #2022–4356-IUGS) and corresponds to the initial experimental phase described in the registered ClinicalTrials.gov protocol (NCT05161832).

### 2.2 Assessment of kinesiophobia and pain catastrophizing

Kinesiophobia was assessed with the French-Canadian version (TSK-CF) of the Tampa Scale for Kinesiophobia validated in the general population (27). The TSK-CF is a valid and reliable 17-item self-assessment questionnaire using Likert scales ranging from 1 (strongly disagree) to 4 (strongly agree), for a total score varying from 17 (lowest level of kinesiophobia) to 68 (highest level of kinesiophobia). The TSK-CF has been shown to have good psychometric properties with Cronbach's *α* = 0.71 and intraclass correlation coefficient > 0.7 (28, 29).

Pain catastrophizing was assessed with the French-Canadian version of the Pain Catastrophizing Scale (PCS-CF). The PCS-CF is a valid and reliable questionnaire comprising 13 items evaluated on Likert scales ranging from 0 (not at all) to 4 (all the time), grouped in 3 dimensions: rumination (cannot help thinking about how much it hurts), amplification (wondering if their pain is severe), and feeling of helplessness (not feeling able to relieve pain). The PCS-CF has been shown to have good psychometric properties with Cronbach's *α* = 0.87 and intraclass correlation coefficient >0.7 (30, 31). The total score is obtained by summing the values of the 13 items and ranges between 0 and 52.

### 2.3 Descending inhibitory pain mechanisms (CPM response)

Evaluation of inhibitory pain mechanisms was conducted using the CPM testing procedure inspired by that of Yarnitsky et al. (32) and described in our previously published studies, and employing a cold pressor test (CPT) and a thermode (30 × 30 mm, TSA-II, Medoc Advanced Medical System, Israel) (26, 33). Participants first underwent a familiarization period with the thermode and the computerized visual analog scale (CoVAS) was used to assess pain. A pretest was then performed to determine the thermode temperature required to induce a moderate pain level of 50/100 (0 = “no pain”, 100 = “worst pain imaginable”) for each participant. This was done by gradually increasing the thermode temperature from 32 °C to 52 °C (0.3 °C/s), while participants rated their pain using the CoVAS.

Once the target temperature had been identified, the first nociceptive test stimulus was applied to the participants’ right forearm at a constant temperature (determined during the pretest) for 2 min. Subjects were told that thermode temperature could increase, remain stable, or decrease over the course of the stimulation, and they were instructed to continuously record their pain level using the CoVAS. Subsequently, a conditioning stimulus (CPT) was applied by immersing the participant's left forearm in cold water at 10 °C for 2 min. Immediately after the CPT, the thermode was reapplied to the right forearm for 2 min, slightly offset from the site of the first stimulation, using the same temperature (second nociceptive test stimulus), during which participants again rated pain intensity on the CoVAS.

In accordance with the guidelines set forth by the European Pain Federation (EFIC®), magnitude of CPM was calculated as the difference between the average CoVAS recorded during the 2 min *after* the CPT and the average CoVAS recorded during the 2 min *before* the CPT. Hence, a negative value denotes pain inhibition (CPM response), while a positive value indicates pain facilitation (34).

### 2.4 Descending motor pathways excitability (slopes, S_50_ and plateaus of IO curves)

Participants were seated comfortably in an armchair with their forearms placed on armrests and their head supported by a headrest. Monophasic magnetic pulses were delivered with a 70 mm figure-eight coil connected to a Magstim 200^2^ stimulator (Magstim Co., Dyfed, UK). The coil was held tangentially to the skull over M1 of the left hemisphere corresponding to the hand area, and the handle oriented at 45° in the medial sagittal plane to induce a posterior to anterior electric current. A neuronavigation system (Brainsight, Rogue Research Inc., Montreal, QC, Canada) was used to ensure consistent position of the TMS coil. The peak-to-peak amplitude of the motor evoked potentials (MEPs) of the right first dorsal interosseus (FDI) and anterior deltoid (AD) muscles were recorded using surface silver EMG electrodes (27 × 37 × 13 mm, inter-electrode spacing 10 mm; Delsys Trigno™, Delsys Incorporated, MA 01760, USA) over the muscle's belly. Electromyographic signals were amplified (×1000) and band-pass filtered (10–1000 Hz) before being digitally sampled at 2 kHz using a 1401 Micro MKII device (Cambridge Electronic Design, Cambridge, UK). To facilitate MEP acquisition for the AD muscle and ensure comparability between the AD and FDI (35, 36), both muscles were recorded while the participant maintained a steady isometric contraction at 8%–12% of the maximal voluntary contraction for each muscle using visual live feedback cursor on a screen in front of them. Contraction of the AD was elicited via shoulder flexion with the elbow fully extended, typically requiring only a slight arm lift, whereas FDI activation was achieved through an index finger-thumb pinch movement, amounting to a light and gentle grip. A 1401 + interface (Cambridge Electronic Device, Cambridge, UK) equipped with Signal software (Cambridge Electronic Design, Cambridge, UK) was used for recording and displaying live to the participant the root mean square (RMS) electromyography (EMG) level of the low-passed (100 Hz) signal, expressed as a percentage of maximal voluntary contraction EMG.

TMS acquisition was aimed at obtaining IO curves, depicting the relationship between TMS stimulus intensity and the resulting motor system response as measured by MEP amplitude (37, 38). The IO curve reflects how quickly and efficiently the nervous system recruits additional neurons in response to increasing stimuli, characterized by 3 parameters: the S_50_, which reflects CSE; the slope, which reflects the recruitment rate (38–40); and the plateau (maximum corticospinal output or saturation), which reflects maximum activation capacity. A lower S_50_ indicates higher sensitivity, meaning that a lower intensity of stimulus is needed to elicit a MEP response, and vice versa. For its part, an increase in slope indicates a higher recruitment rate, meaning that a greater number of neurons are activated with smaller increases in stimulus intensity. Finally, a high plateau in the TMS I/O curve reflects an increased capacity for maximal corticospinal output, likely due to heightened excitatory drive or reduced inhibitory control, and may indicate a system with robust adaptability or plasticity (38). To build the IO curve, at least 8 MEPs were recorded at each stimulus intensity, ranging from subthreshold intensity (3% beneath threshold) to that evoking the largest response or until maximum stimulator output (MSO) was reached. These acquisitions were done in an incremental order, with steps of 3 to 5% of the magnetic stimulator output (41). The average peak-to-peak MEP amplitude was plotted against the stimulus intensity, and the Levenberg-Marquard nonlinear least-mean-squares algorithm of GraphPad Prism (version 9.0.0) (42) was used to fit the data points to a Boltzmann sigmoidal equation. This equation (1) relates MEP amplitudes to stimulus intensity (S) as follows:

$$MEP(S)=y_{0}+\frac{MEP_{MAX}}{1+e^{\frac{S_{50}-S}{k}}}$$ (1)

This equation has four parameters:

1. MEP_MAX_ corresponds to the maximum corticospinal output or the plateau of the IO curve;
2. S_50_ is the stimulus intensity required to obtain 50% of the maximum response;
3. k, and its reciprocal (i.e., 1/k) is directly proportional to the maximum slope of the curve, which occurs at S_50_;
4. y_0_ corresponds to the floor of the IO curve.

For this study, the slope of the IO curve and the S_50_ were the key metrics, providing a comprehensive index of CSE (43, 44).

The TMS assessment was carried out on four occasions, the first two times under pain-free conditions for the FDI then for the AD muscle, and the last two after the onset and stabilization of experimental pain for the FDI and AD muscles.

### 2.5 Experimental pain

Experimental pain was induced using ∼0.1 ml of a topical 1% capsaicin cream, applied on the participant's right delto-pectoral groove, on intact non-irritated skin (19, 45). A warm moist pad was applied to the site to promote capsaicin absorption and accelerate the onset of pain (46). Pain was assessed at regular intervals, every 1–2 min, ensuring a minimum pain intensity of 3/10 on the visual analogue scale (VAS; 0 = “no pain”, 10 = “worst pain imaginable”). Pain stabilization, defined as 2 consecutive identical pain measurements on the VAS, was confirmed prior to starting the second TMS assessment (pain condition).

### 2.6 Statistical analysis

Statistical tests were performed using JASP software (version 0.95.4). Because Shapiro–Wilk tests and visual inspection revealed the data were not normally distributed, nonparametric tests were used for all analyses. First, to assess whether baseline maximum corticospinal output prior to pain exposure was associated with the magnitude of the CPM responses, a Spearman's correlation analysis was conducted between CPM responses and the plateau values in the pain-free condition. Second, Spearman's correlation analyses were performed to determine if CPM responses correlated with pain-induced changes in IO curves’ slopes and S_50_ values for AD and FDI muscles. These changes were calculated by subtracting pain-free condition values from painful condition values, yielding positive values for increases and negative values for decreases. According to the classification of Mukaka (47), correlation coefficients were interpreted as follows: 0.9 to 1 = very highly correlated; 0.7 to 0.9 = highly correlated; 0.5 to 0.7 = moderately correlated; 0.3 to 0.5 = weakly correlated; and 0 to 0.3 = negligible. Finally, participants were dichotomized according to median scores to examine the effects of kinesiophobia and pain catastrophizing, as parametric modeling was not feasible; correlation coefficients were subsequently compared between groups. This allowed us to assess the relationships between CPM and TMS metrics for each group, and to compare the strength of these associations using Fischer r-to-z tests. In an attempt to enhance replicability, minimize false positives results, and uphold scientific rigor, we embraced the changes proposed by Benjamin et al. wherein the threshold for statistical significance is lowered to *p* < 0.005 and *p*-values ranging between 0.05 and 0.005 are considered “suggestive” (48).

## 3 Results

### 3.1 Participants and experimental induced pain

Of the 21 participants recruited, one did not complete the evaluation (unable to understand the instructions for the CPM protocol) and was excluded from the analyses. The remaining 20 participants all completed the assessments and were included in all the analyses (see Figure 1). The characteristics of the participants are summarized in Table 1. After pain stabilization, participants reported a mean capsaicin-induced pain intensity of 6.9/10 on the VAS, which represents both a statistically (*p* < 0.001) and clinically (49, 50) significant increase compared to the baseline (no pain) condition.

**Table 1:** Participants’ characteristics.

Sample characteristics	Number or mean (SD)
Sex (F/M)	10/10
Age (years)	36.8 (16.8)
Height (m)	1.7 (0.1)
Weight (kg)	78.0 (17.1)
Ethnic group (Caucasian/Black/Hispanic)	17/2/1
Kinesiophobia (TSK)	36.1 (8.3)
Pain Catastrophizing (PCS)	13.6 (11.3)

SD, standard deviation; F/M, female/male; TSK-CF, Tampa scale for kinesiophobia; PCS, Pain catastrophizing scale.

**Figure 2:**
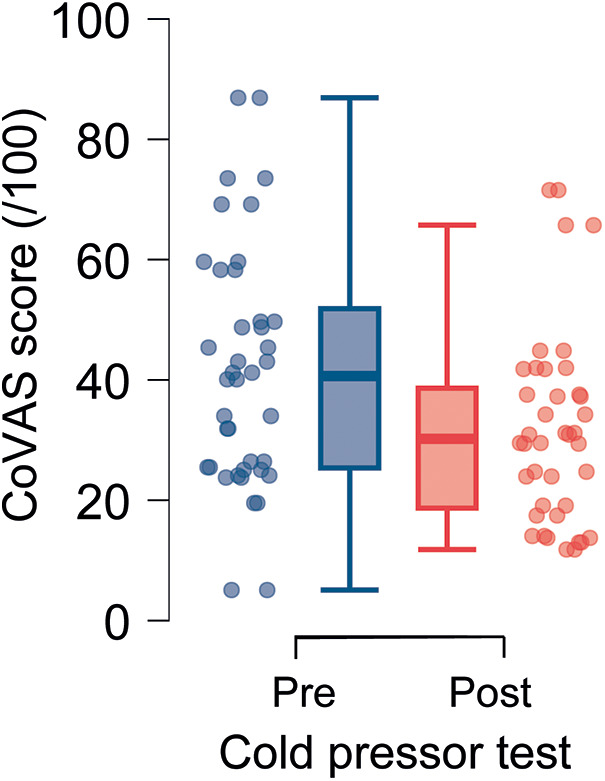
Box plot with scatter of the CoVAS mean score of pain rate before and after cold pressor test (CPT).

Participants rated the pain intensity of the CPT at an average of 5.4 (±3.3) on a 10-point pain rating scale. The mean pain experienced by the participants during the 2-min thermode test (test stimulus) before and after the CPT is illustrated in Figure 2. As depicted in this figure, thermode-induced pain was, on average, smaller following the conditioning CPT stimulus compared to pain levels reported before CPT, confirming that the conditioning stimulus activated descending pain inhibitory mechanisms.

**Figure 3:**
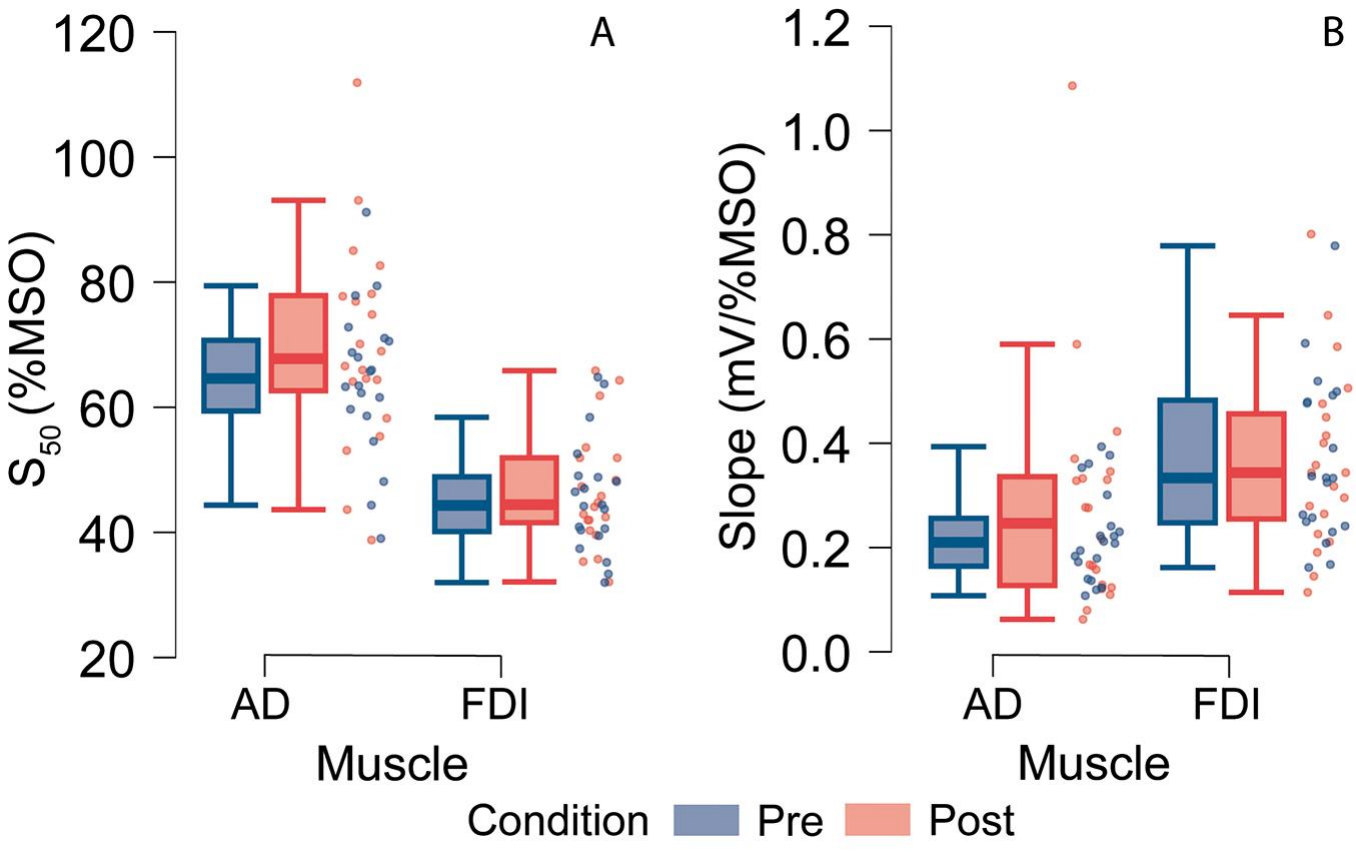
Box plot with scatter of the corticospinal excitability measurements for the anterior deltoid and first dorsal interosseous before and after the application of capsaicin cream, presented for both S_50_ **(A)** and IO curve slopes values **(B).**

The effect of pain on TMS measurements is illustrated in Figure 3. As can be seen in this figure, pain resulted in an increase in S_50_ for AD (63.5 to 69%MSO) and, to a lesser extent, for FDI (45.8 to 46.5%MSO), and a slight increases in IO curve's slopes for AD (0.22 to 0.27 mV/%MSO) and FDI (0.35 to 0.36 mV/%MSO).

### 3.2 Relationships between descending inhibitory pain mechanisms (CPM response) and corticospinal excitability (IO slopes, S_50_ and plateaus)

**Figure 4:**
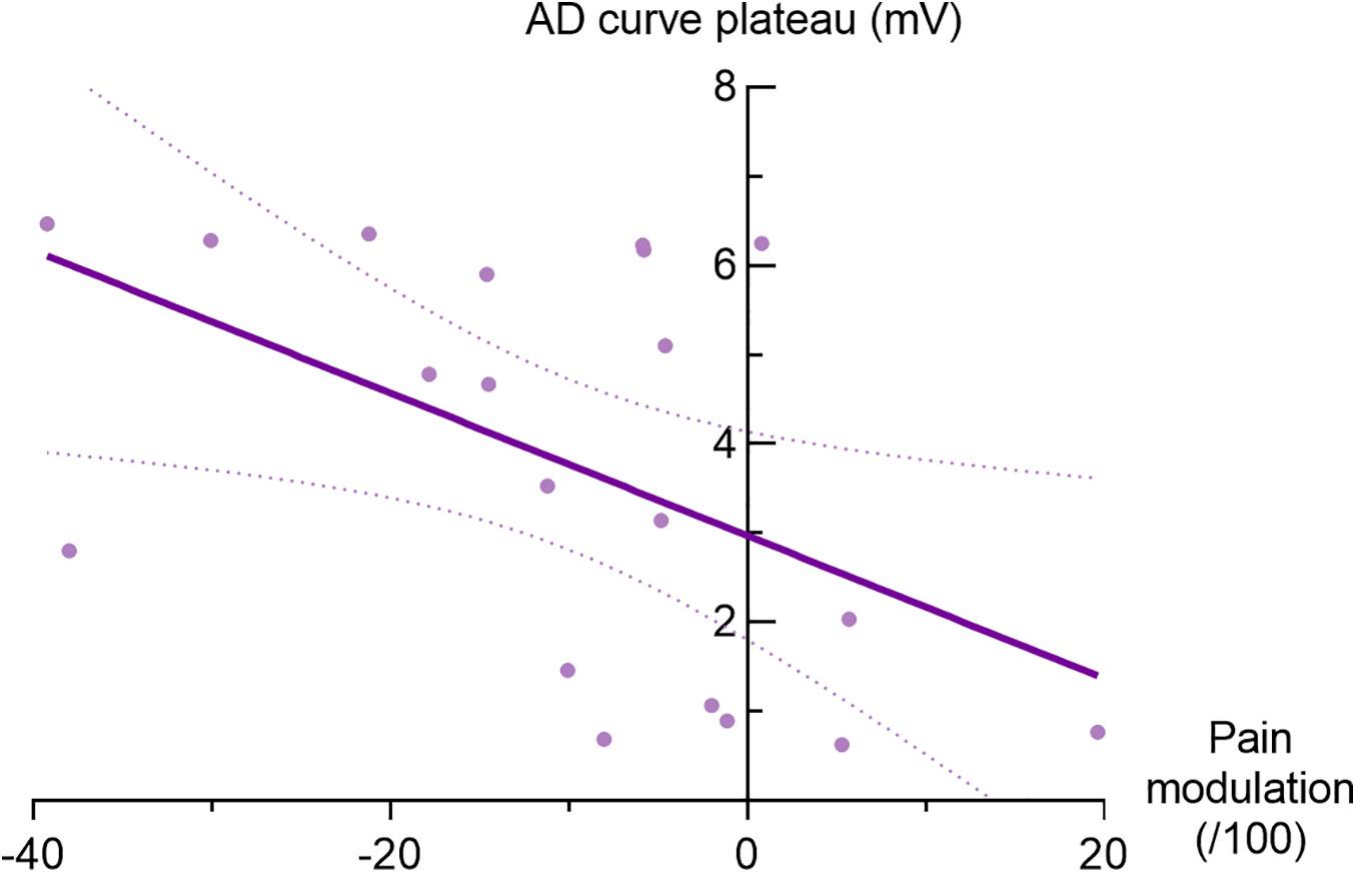
Correlation between CPM responses and plateau of IO curve for the anterior deltoid muscle. Purple line represents regression line and dotted line represents 95% confidence interval (r_S_ = −0.56, *p* = 0.01).

Spearman correlation analyses were conducted to evaluate the relationship between CPM responses and TMS measures. For the AD muscle, a moderate and negative correlation was found between the baseline plateau of the IO curve and CPM responses (r_s_ = −0.56, *p* = 0.01; see Figure 4), indicating that greater maximal corticospinal output of the corticospinal pathway was associated with more effective endogenous pain inhibition. No significant correlations were observed between CPM responses and pain-induced variations in the IO slope (*p* = 0.49) or S_50_ (*p* = 0.76).

**Figure 5:**
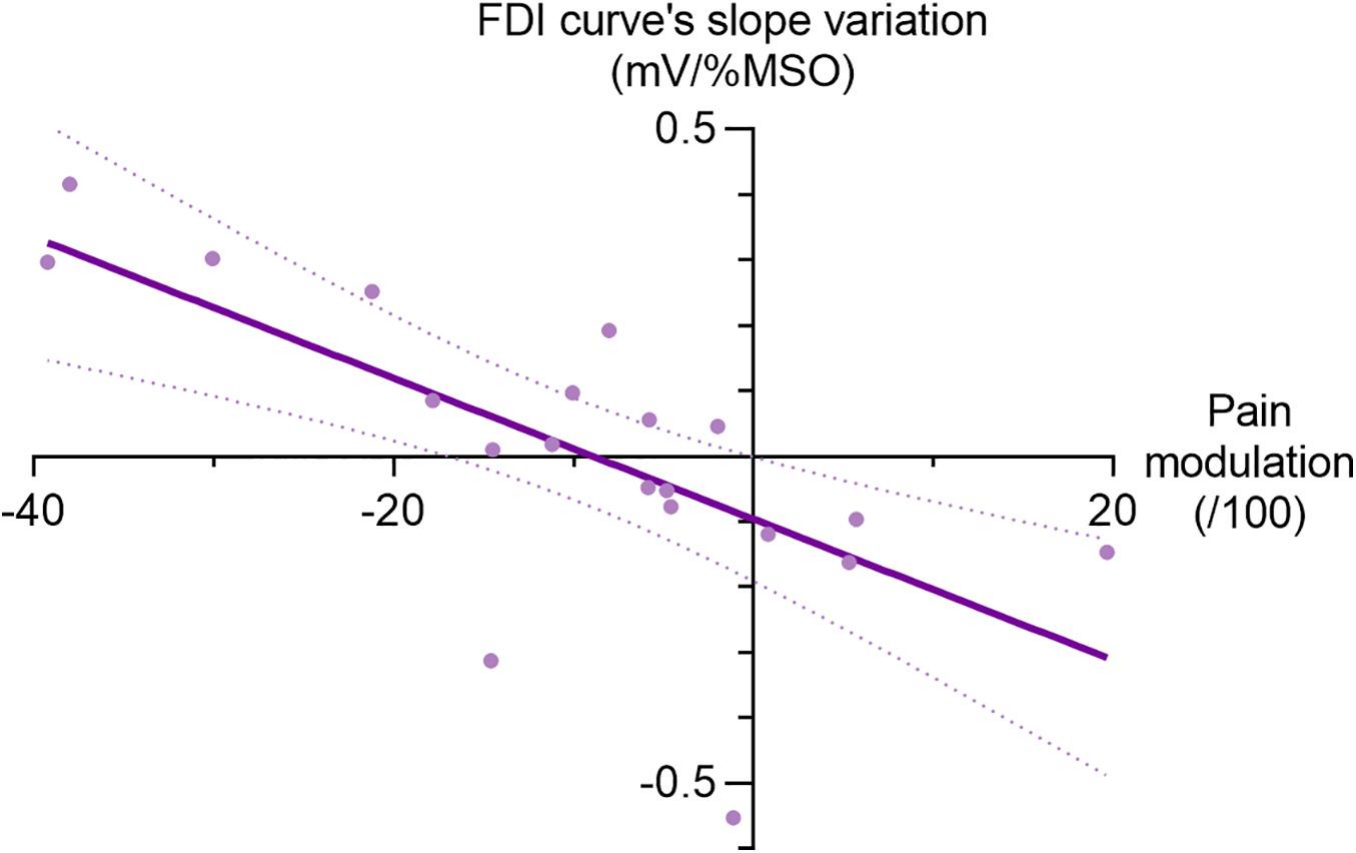
Correlation between CPM responses (delta score) and pain-induced changes in IO slopes for the first dorsal interosseous muscle. Purple line represents regression line and dotted line represents 95% confidence interval (r_S_ = −0.75, *p* < 0.001).

For the FDI muscle, a strong and negative correlation was observed between CPM responses and pain-induced IO slope variations (r_s_ = −0.75, *p* < 0.001; see Figure 5), suggesting that individuals with more effective endogenous pain inhibition exhibit greater increases in IO slope under pain conditions. No significant correlations were found between CPM responses and the baseline plateau of IO curves (*p* = 0.90) or pain-induced S_50_ variations (*p* = 0.57).

### 3.3 Influence of kinesiophobia and pain catastrophizing on the relationship between IO curve and CPM response

The correlation coefficients between CPM and TMS measures for individuals with low and high TSK and PCS scores (determined by median-split) are reported in Tables 2 and 3, respectively. The Fisher r-to-z analysis revealed the presence of a statistically suggestive difference between participants with high and low TSK scores for pain-induced S_50_ variations for the AD muscle (*p* = 0.02), indicating that the variations of S_50_ was differently associated with CPM responses for individuals with low and high kinesiophobia. This difference was also reflected in the direction of the association, shifting from a positive correlation (low TSK scores) to a negative (high TSK scores) correlation. No TSK between-group difference was found for the FDI muscle, and for the PCS scores (AD and FDI).

**Table 2:** Comparison of spearman correlations between CPM responses and corticospinal excitability among lowest and highest TSK scores.

Variable	CPM		*p* value
	Lowest TSK (*n* = 10)	Highest TSK (*n* = 10)	
First dorsal interosseous			
Delta IO curves’			
Slope (mV/%MSO)	−0.84	−0.67	0.22
S_50_ (%MSO)	0.32	−0.08	0.22
Anterior deltoid			
Delta IO curves’			
Slope (mV/%MSO)	−0.24	0.08	0.27
S_50_ (%MSO)	0.43	−0.59	**0** **.** **017**

CPM, conditioned pain modulation; TSK, Tampa Scale for Kinesiophobia; IO, input output; MSO, maximum stimulator output.

Bold text means *p* < 0.05.

**Table 3:** Comparison of spearman correlations between CPM responses and corticospinal excitability among lowest and highest PCS scores.

Variable	CPM		*p* value
	Lowest PCS (*n* = 10)	Highest PCS (*n* = 10)	
First dorsal interosseous			
Delta IO curves’			
Slope (mV/%MSO)	−0.75	−0.72	0.46
S_50_ (%MSO)	0.30	−0.01	0.28
Anterior deltoid			
Delta IO curves’			
Slope (mV/%MSO)	0.12	−0.50	0.11
S_50_ (%MSO)	−0.08	−0.13	0.46

CPM, conditioned pain modulation; PCS, Pain catastrophizing scale, IO, input output; MSO, maximum stimulator output.

## 4 Discussion

The aim of this study was to explore the potential association between descending motor pathways and descending pain inhibitory pathways, and to determine whether kinesiophobia or pain catastrophizing could influence this relationship. Our findings reveal that CPM responses were significantly correlated with the baseline IO plateau of the AD muscle, located proximally to the nociceptive stimulus, suggesting a pre-existing state of CSE may influence descending pain modulation. Furthermore, CPM responses were associated with pain-induced changes in the IO slope of the FDI muscle, located distal to the pain site, indicating that nociceptive input can modulate excitability in remote motor representations. Finally, our results suggest a potential divergence in the direction of the association between CPM responses and pain-induced changes in S_50_ for the muscle proximal to the pain site (AD), depending on individuals’ levels of kinesiophobia.

### 4.1 Interaction between pain modulation and corticospinal excitability

Participants who presented the highest baseline plateaus for AD in the pain-free condition were those with the most effective endogenous pain inhibition responses. These results further extend upon the observations of Granovsky et al. and Martel et al., who reported a correlation between effective CPM responses and increased baseline CSE, both in pain-free individuals and in those with chronic pain. The results obtained by Granovsky et al. and Martel et al. were based exclusively on static assessments of CSE under baseline pain conditions, using a single TMS intensity, and did not address the dynamic adaptability of the IO curves to acute pain. In contrast, our study employed a dynamic approach by measuring CSE in response to experimentally induced pain. This approach revealed that, for the FDI muscle (located distal to the painful site), approximately half of the participants exhibited increased excitability, while the other half showed a decrease. It is particularly striking and noteworthy to observe that this association between the pain-induced changes in IO curve's slope and CPM responses was present for a muscle located at a distant, pain-free site (FDI), rather than for a muscle surrounding the painful site (AD).

These unexpected findings could potentially be explained by the fact that pain-induced modulation of CSE varies depending on the muscle's capacity for compensation and its anatomical proximity to the painful site. Such differences could facilitate the implementation of alternative motor synergies or strategies (51, 52). For instance, the AD muscle may require stable CSE to ensure effective shoulder flexion, a task for which compensation is challenging (53). In contrast, the hand comprises multiple small muscles capable of compensating for one another, thereby allowing a greater variability in CSE (54).

Regarding MEP in relation to nociception, similar findings have been reported in individuals without chronic pain. For instance, a study investigating the effects of intramuscular electrical stimulation for neck muscles in patients with chronic pain found that higher baseline MEP amplitudes were inversely correlated with pain severity after the intervention (55). In another study comparable to ours, active MEP recorded from the AD at intensities near the plateau were significantly higher in healthy individuals than in patients with chronic rotator cuff tears (35). These phenomena could be partly explain by variations in GABA and glutamate neurotransmission (44), potentially influenced by brain-derived neurotrophic factor (BDNF) (56), whereby higher MEP amplitude could reflect a more efficient activation of top-down pain inhibitory mechanisms. This link between pain modulation and motor systems is supported by several neuroanatomical observations. For example, functional connectivity studies have shown that regions involved in endogenous analgesia – such as the periaqueductal gray – are functionally connected to the motor cortex, particularly in contexts of fear and threat (57–59). The close anatomical proximity between the dorsolateral funiculus (which conveys pain inhibitory projections) and the adjacent corticospinal tract within the dorsolateral part of the spinal cord also lends a certain anatomical plausibility to the associations observed (60).

Considering that less efficient CPM in early phase of pain has been associated with pain chronification (11, 61, 62), it would be particularly informative to compare the correlations between CPM responses and CSE in the healthy population and individuals with chronic pain.

### 4.2 Impact of kinesiophobia on S_50_ pain-induced changes

Comparison of correlations between pain-induced changes in CSE and CPM response revealed a difference in the direction of the association for the AD muscle between individuals with low and those with high levels of kinesiophobia. For individuals with the lowest TSK scores, a pain-induced decrease in CSE (positive S_50_ delta score) was associated with reduced endogenous pain inhibition, whereas the opposite (more efficient pain inhibition in individuals with the greatest decrease CSE) was observed in participants with the highest TSK scores.

The interaction between kinesiophobia and pain-induced changes in CSE has been documented previously among pain-free subjects (15, 16). In these studies, low kinesiophobia was associated with decreased IO curve's slope during pain (16) and decreased MEP amplitude after pain resolution (15); conversely, individuals with high levels of kinesiophobia showed no change in CSE. Considering the impact of cognition on CPM response (63), it is plausible that the level of kinesiophobia could play a role in complex interactions between pain, pain modulation and certain parameters of CSE in specific muscles (such as S_50_ for the FDI) by interacting with brain regions involved in the emotional and affective processing of pain. These regions encompass the cortico-limbic pain circuit, including the prefrontal cortex, anterior cingulate cortex, amygdala, and nucleus accumbens (64, 65). Further studies are needed to confirm the involvement of kinesiophobia in this relationship, to understand the direction of the correlations, and to identify the specific aspects of CSE and muscles involved.

Our findings that pain enhances hand CSE in individuals with more efficient CPM responses could provide a mechanistic framework for understanding why excitatory rTMS (≥5 Hz) may be effective in chronic pain, and help clinicians identify patients who are more likely to respond to this type of intervention. By stimulating M1 and transiently enhancing CSE, these protocols could help engage the endogenous inhibitory systems that mediate CPM responses, which could be particularly useful in patients with altered inhibitory pain mechanisms.

### 4.3 Strengths and limits

To the best of our knowledge, this study represents the first attempt to establish correlations between endogenous pain modulation and the adaptation of CSE to experimentally induced pain, while taking into considerations psychological factors such as kinesiophobia and pain catastrophizing. Unlike the prevailing focus of previous studies, we investigated CSE adaptations in muscles both proximal and distal to the painful site.

This study has certain limitations, the most significant being the small sample size, which increases the risk of committing type II errors. Moreover, the absence of normal data distribution prevented the use of parametric tests, constraining the analyses. Non-significant correlations should thus be interpreted with caution. Furthermore, while capsaicin offers many methodological advantages (e.g., non-invasiveness, controlled dosage and stability in pain ratings) (66, 67), it fails to reproduce the variety of symptoms presented by individuals suffering from clinical pain conditions.

Future research should also investigate the relationship between CPM and distant or contralateral muscles, as well as the role of attention in pain-induced CSE modulation.

## 5 Conclusions

This study provides novel evidence that CSE responses to pain are closely linked to individual variability in endogenous pain modulation. Specifically, individuals with higher baseline maximum corticospinal output for the AD also exhibited stronger CPM responses, indicating that static properties of the motor system may reflect the functional status of endogenous modulation even prior to nociceptive challenge. Higher pain-induced facilitation of FDI corticospinal output also exhibited more efficient CPM, suggesting that effective descending pain inhibition could be linked to dynamic CSE adaptation during pain. Importantly, psychological factors such as kinesiophobia appeared to modulate these associations, highlighting the interplay between motor system adaptation, pain modulation, and affective-motivational processes. These findings suggest that the motor system's response to pain cannot be fully understood without considering both neurophysiological and psychological contributions. They also support the notion that corticospinal responses to pain may serve as indirect markers of endogenous pain inhibitory function, with potential implications for identifying individuals at risk for maladaptive pain processing. Future research should further explore these relationships in clinical populations to determine their prognostic or therapeutic relevance.

## Data Availability

The raw data supporting the conclusions of this article will be made available by the authors, without undue reservation.
